# A Scalable Manufacturing Approach to Single Dose Vaccination against HPV

**DOI:** 10.3390/vaccines9010066

**Published:** 2021-01-19

**Authors:** Shuai Shao, Oscar A. Ortega-Rivera, Sayoni Ray, Jonathan K. Pokorski, Nicole F. Steinmetz

**Affiliations:** 1 Department of NanoEngineering, University of California San Diego, La Jolla, CA 92093, USA; lovefavor0509@hotmail.com (S.S.); oortegarivera@eng.ucsd.edu (O.A.O.-R.); saray@eng.ucsd.edu (S.R.); 2 Center for Nano-ImmunoEngineering, University of California San Diego, La Jolla, CA 92093, USA; 3 Institute for Materials Discovery and Design, University of California San Diego, La Jolla, CA 92093, USA; 4 Department of Bioengineering, University of California San Diego, La Jolla, CA 92093, USA; 5 Department of Radiology, University of California San Diego, La Jolla, CA 92093, USA; 6 Moore’s Cancer Center, University of California-San Diego, La Jolla, CA 92093, USA

**Keywords:** HPV vaccine candidate, L2 protein, Qβ, virus-like particles (VLPs), PLGA implants, vaccine delivery device, hot melt extrusion

## Abstract

Human papillomavirus (HPV) is a globally prevalent sexually-transmitted pathogen, responsible for most cases of cervical cancer. HPV vaccination rates remain suboptimal, partly due to the need for multiple doses, leading to a lack of compliance and incomplete protection. To address the drawbacks of current HPV vaccines, we used a scalable manufacturing process to prepare implantable polymer–protein blends for single-administration with sustained delivery. Peptide epitopes from HPV16 capsid protein L2 were conjugated to the virus-like particles derived from bacteriophage Qβ, to enhance their immunogenicity. The HPV-Qβ particles were then encapsulated into poly(lactic-co-glycolic acid) (PLGA) implants, using a benchtop melt-processing system. The implants facilitated the slow and sustained release of HPV-Qβ particles without the loss of nanoparticle integrity, during high temperature melt processing. Mice vaccinated with the implants generated IgG titers comparable to the traditional soluble injections and achieved protection in a pseudovirus neutralization assay. HPV-Qβ implants offer a new vaccination platform; because the melt-processing is so versatile, the technology offers the opportunity for massive upscale into any geometric form factor. Notably, microneedle patches would allow for self-administration in the absence of a healthcare professional, within the developing world. The Qβ technology is highly adaptable, allowing the production of vaccine candidates and their delivery devices for multiple strains or types of viruses.

## 1. Introduction

Human papillomavirus (HPV) is a globally-prevalent pathogen and the most common sexually-transmitted infection in the USA [1]. There are many strains of HPV, including low-risk types associated with warts, and high-risk types that give rise to various forms of anal, penile, and oropharyngeal cancer, as well as almost all cases of cervical cancer [2]. Most HPV infections cause no symptoms and are cleared by the immune system, but persistent infections might lead to the more serious health issues listed above [1]. Vaccines can protect against the most dangerous strains of HPV, and three were approved [3]. The latest (Gardasil 9) protects against nine HPV types and prevents 90% of HPV-associated cancers every year [2,4,5,6].

Despite the protection offered by the HPV vaccine, only 53.7% of girls and 48.7% of boys in the US were vaccinated against HPV in 2019 [7]. This compares poorly to 66% of girls and 42% of boys who completed the vaccination program in 2011, suggesting that take-up rates are declining [8]. The poor quality of public health services in many developing countries means the vaccination rates are even worse, even though HPV infections are more prevalent [9]. Factors that contribute to the poor acceptance of the HPV vaccine include costs, lack of knowledge about HPV transmission, parental distrust of vaccines, and the prolonged vaccination schedule (which requires three injections to achieve full protection). Based on 2011 data, only 70.7% of girls and 28.1% of boys who receive the first dose go on to complete the course [8,10]. Another considerable logistical and fiscal barrier is the cold chain requirement for HPV vaccines, making it untenable to distribute life-saving vaccines in resource-poor areas of the world. Innovating vaccine platforms and delivery devices to break cold chain limitations is therefore an excellent solution to safeguard potent vaccination for both wealthy and lower-income countries. 

The HPV capsid is composed of two proteins—the major capsid protein L1, which forms the virus structure, and the minor capsid protein L2, which is required for genome encapsidation and infection [11,12,13]. All three approved HPV vaccines comprise virus-like particles (VLPs) that spontaneously self-assemble from protein L1, which is immunogenic and can elicit high titers of HPV-specific antibodies. However, one limitation of L1 is that the vaccines are type-specific unless they contain multiple VLPs representing each target strain. Accordingly, Gardasil 9 achieves protection against nine types of HPV because it contains nine different VLPs [14]. In contrast to L1, the immunogenicity of protein L2 is low, probably because it is only transiently displayed on the virion surface during infection [15]. However, some epitopes of L2 are highly conserved in diverse HPV types, and HPV vaccines based on L2 epitopes can therefore elicit broadly-neutralizing antibodies that protect against a wide range of HPV types [16,17]. L2 does not assemble into VLPs, therefore vaccines based on this protein must incorporate it into a carrier. Various nanoparticles were developed for this purpose because it is easy to control their physicochemical properties (e.g., size, shape, and hardness) and they can be modified to display multiple copies of the epitope, enhancing their immunogenicity [18,19,20]. 

Challenges remain to produce vaccine candidates that are stable without the cold chain, to be distributed worldwide. Further, the ideal candidate would be delivered using a vaccine delivery device, such as a microneedle, enabling self-administration in the absence of a healthcare professional. Toward this goal, we set out to develop a process to produce implants for sustained release of HPV vaccine candidates. Such delivery devices hold the potential to overcome the need for repeat dosing. Biodegradable polymers are ideal slow-release carriers for antigens, as sustained exposure to antigens over time leads to an improved immunological response [21,22,23,24]. We recently described a melt-processing method to encapsulate VLPs into biodegradable polymers and when implanted intact VLPs were released slowly over time [25]. The advantage of melt-processing to develop slow-release implants is its massive scalability, reproducibility, and solvent-free nature. One reason that proteins can undergo the high temperatures necessary for melt-encapsulation, is the reduced hydration state, which enhances thermal stability. Furthermore, VLPs are able to undergo the rigors of melt-processing due to their exceptional stability, with negligible degradation or aggregation at temperatures of ~100 °C. When the implants were loaded with immunogenic VLPs and implanted subcutaneously, the resulting antibody titers were similar to the traditional schedule with three doses, providing confidence that the implants could be used as a single-dose vaccine platform. 

Here, we developed a single-dose HPV vaccine in which L2 peptide antigens from the high-risk HPV16 strain were displayed on the surface of VLPs derived from bacteriophage Qβ, a carrier used in several other vaccine candidates [26,27]. We chose a bioconjugate chemistry protocol to achieve multivalent presentation of L2 antigens on the Qβ carrier. A combination of size exclusion chromatography (SEC), transmission electron microscopy (TEM), and dynamic light scattering (DLS), and gel electrophoresis was performed to validate the structural integrity and epitope display of the HPV-Qβ. Immunogenicity was validated in healthy mice and the antibody titers and subtypes were determined using the ELISA-based protocols. The HPV-Qβ particles were then encapsulated in poly(lactic-co-glycolic acid) (PLGA) implants, using a benchtop melt-processing system, and implanted into mice. We monitored the release rate of the VLPs in vitro and in vivo and confirmed that the HPV-Qβ particles released remained intact. Finally, immunizations were carried out by comparing the implant vs. soluble injections. Data showed that the antibody titers generated by the single-dose HPV-Qβ/PLGA implant were equivalent to traditional injection schedules and played a neutralizing role against the HPV pseudovirus. 

## 2. Materials and Methods

### 2.1. Expression of Qβ

Bacteriophage Qβ VLPs were expressed and purified, as previously described [28]. Chemically competent BL21(DE3) *Escherichia coli* cells (New England Biolabs) transformed with pET28CP containing the Qβ coat protein sequence were plated onto lysogeny broth (LB) agar (Thermo Fisher Scientific) containing 50 μg/mL kanamycin (Gold Biotechnology). The isolated colonies were picked into 100 mL of autoclaved LB medium plus kanamycin and cultured for 12 h at 37 °C to saturation. We added 50 mL of the culture to 1 L MagicMedia (Thermo Fisher Scientific, Carlsbad, CA, USA) and incubated at 37 °C for another 24 h, shaking at 300 rpm. The cell pellets were collected by centrifugation at 1500× *g* and frozen at −80 °C overnight. The pelleted cells were resuspended in 100 mL phosphate-buffered saline (PBS) on ice and lysed using a probe sonicator for 10 min. The lysate was centrifuged at 10,080× *g*, the supernatant was collected, and the Qβ particles were precipitated by adding 10% *w*/*v* PEG8000 (Thermo Fisher Scientific) at 4 °C for 12 h on a rotisserie. The precipitated fraction was pelleted by centrifugation at 10,080× *g* and dissolved in 40 mL PBS before extraction with a 1:1 v/v butanol/chloroform. The aqueous fraction was collected by centrifugation as above (at 10,080× *g*) and Qβ particles were purified on a 10–40% sucrose velocity gradient by ultracentrifugation at 96,281× *g* for 4.5 h. The light-scattering Qβ layer was collected and pelleted by ultracentrifugation at 160,326× *g* for 3 h. The purified Qβ particles were dissolved in PBS as a stock solution for later experiments.

### 2.2. Synthesis of HPV-Qβ

HPV peptides were conjugated to Qβ via an SM(PEG)_8_ bifunctional linker containing an NHS group and a maleimide group (Thermo Fisher Scientific). In brief, 20 mg of Qβ was mixed with a 500-fold molar excess of SM(PEG)_8_ to Qβ in 0.5 mL PBS (pH 7.4), at room temperature for 1 h. The unreacted SM(PEG)_8_ was removed using a 100 kDa cut-off centrifuge filter at 3000× *g* for 15 min, and the recovered VLPs were washed with 0.5 mL PBS. The washed VLPs were resuspended in 0.5 mL PBS and reacted with a 500-fold molar excess of HPV L2 peptides at room temperature for 2 h. The unreacted peptides were removed by centrifugal filtration as above; the recovered HPV-Qβ vaccine candidates were washed twice in 0.5 mL deionized water, and then dialyzed against deionized water for 24 h. 

### 2.3. Synthesis of Qβ-Cy5

Bacteriophage Qβ (20 mg) was labeled with Cy5 through conjugation via a 500-fold molar excess of sulfo-Cy5 NHS ester (Lumiprobe) in 0.5 mL 0.1 M potassium phosphate buffer (referred to as KP buffer; K_2_HPO_4_ and KH_2_PO_4,_ pH 8.3), for 4 h at room temperature. The unreacted components were removed by centrifugal filtration as above, and the Cy5-Qβ particles were dialyzed against and then stored in deionized water.

### 2.4. Preparation of Qβ-Loaded PLGA Implants through Melt-Extrusion

The loaded PLGA implants were prepared using a new desktop melt-processing system [29]. EXPANSORB PLGA (PCAS, 50:50, MW~20,000 Da) and FITC-PLGA (Akina) were ground to a fine powder. The Qβ, HPV-Qβ, or Cy5-Qβ particles were lyophilized before melt extrusion. The formulation of all implants was 80% PLGA, 10% VLP, and 10% PEG8000. The powdered components were mixed in a 2-mL centrifuge tube by vortexing for 20 min, then loaded into the melt-processing system (maximum load = 60 mg) and heated to 70 °C for 90 s. The air pressure was gradually increased up to 10 psi to extrude the implant cylinders, which were dried for 1 h and cut into 0.3–0.5 cm lengths, according to the weight.

### 2.5. Implant Analysis by SEM-EDX

Cross-sections of the implants were coated with carbon and observed by scanning electron microscopy with energy dispersive X-ray spectroscopy (SEM-EDX), using an FEI Quanta 600 scanning electron microscope coupled to a Bruker XFlash 6/60 EDX spectroscope. The EDX maps were prepared using the FEI AZtec software.

### 2.6. Characterization of Particles

The VLPs were characterized by fast protein liquid chromatography (FPLC), transmission electron microscopy (TEM), dynamic light scattering (DLS), and sodium dodecylsulfate polyacrylamide gel electrophoresis (SDS-PAGE). FPLC was performed using an AKTA-FPLC 900 system fitted with Superose 6 Increase 10/300 GL columns (GE Healthcare), using PBS (pH 7.4) as the mobile phase at a flow rate of 0.5 mL/min. TEM images were acquired on an FEI Tecnai Spirit G2 Bio TWIN transmission electron microscope. Samples were mounted on 400-mesh hexagonal copper grids and stained with 2% uranyl acetate. DLS was carried out on a Malvern Instruments Zetasizer Nano at 25 °C, in plastic disposal cuvettes. SDS-PAGE was performed on NuPAGE 12% Bis-Tris protein gels (Thermo Fisher Scientific) at 150 mV for 75 min. The gels were stained with Coomassie Brilliant Blue and images were acquired using the ProteinSimple FluorChem R imaging system. 

### 2.7. Analysis of the Loaded PLGA Implants In Vitro

Qβ-loaded PLGA implants (~3 mg implant containing 300 µg VLPs) were placed in a 1.5-mL centrifuge tube and immersed in 500 µL PBS at 37 °C. At each time-point, 200-µL samples were removed and replaced with 200 µL fresh PBS. The quantity of released VLPs was determined by measuring the total protein concentration using a Pierce BCA assay kit (Thermo Fisher Scientific). 

### 2.8. Analysis of Cy5-Qβ Loaded FITC-PLGA Implants In Vivo

All animal experiments were carried out in accordance with recommendations from the UCSD’s Institutional Animal Care and Use Committee (ethical approval: UCSD IACUC protocol S18021). Female BALB/c mice aged 8 weeks (Jackson Laboratory, Bar Harbor, ME, USA) were anesthetized for the implantation process before shaving hair from the back and wiping the skin with Alcohol Prep pads. The FITC-PLGA implants loaded with Cy5-Qβ VLPs (~1 mg implant containing 100 µg VLPs) were introduced subcutaneously via an 18-gauge needle by pushing the implant out of the needle with a sterilized stainless-steel wire (0.51 mm diameter). The needle was withdrawn and the skin wiped with Alcohol Prep pads. Fluorescence images were acquired on an IVIS 200 imaging system at different time-points and were analyzed using Living Image v3.0. 

### 2.9. Immunization

For vaccines in solution, female BALB/c mice aged 8 weeks were vaccinated subcutaneously by injection on days 0, 14, and 28, with blood collected 7 days after each vaccination. For the PLGA implants, mice were vaccinated by subcutaneous implantation, as described above, and blood was collected on days 7, 14, 21, 35, and 49. To compare the different HPV-Qβ conjugates, n = 5 mice were immunized three times with 30 µg of the specific HPV-Qβ conjugate (or the Qβ as a control). For the conjugates vs. mixture vs. peptide experiment, n = 6 mice were vaccinated with 30 µg of the specific HPV-Qβ conjugate, a mixture of 30 µg Qβ and 5 µg of the HPV peptide, or 5 µg of the HPV peptide alone. For the evaluation of single-dose vaccination using the loaded PLGA implants, the PLGA implants were subcutaneously introduced into n = 6 mice. The implants contained 100 µg HPV-Qβ, 500 µg HPV-Qβ, a mixture of 100 µg Qβ, and 20 µg HPV peptide, or 20 µg HPV peptide alone. To make a direct comparison among the three different vaccination techniques, three different groups of mice each comprising n = 6 mice were vaccinated subcutaneously with a single dose of 100 µg of the HPV-Qβ conjugate, three doses of 30 µg of the HPV-Qβ conjugate, and a single dose HPV-Qβ-loaded PLGA implants, loaded at 100 µg. Sera were separated from the collected blood through centrifugation at 2000× *g* for 20 min and were stored at 4 °C (short term) or −80 °C (long term).

### 2.10. Serum Analysis and Determination of Antibody Titers

The abundance of antibodies against HPV peptides and Qβ particles in serum samples was determined by the enzyme-linked immunosorbent assay (ELISA). For the detection of α-HPV antibodies, 96-well Pierce maleimide-activated plates (Thermo Fisher Scientific) were coated with 100 µL HPV peptides (10 µg/mL) per well in coating buffer (0.1 M sodium phosphate, 0.15 M sodium chloride, 10 mM EDTA, pH 7.2). After overnight incubation at 4 °C, the plates were washed three times with PBS containing 0.1% Tween-20 (PBST) and then blocked with 150 µL cysteine (10 µg/mL) per well for 1 h, at room temperature. After three washes in PBST, serial dilutions of serum in PBST containing 1% bovine serum albumin (BSA; Roche Diagnostics, Mannheim, Germany) were added and incubated at 37 °C for 1 h. After three washes in PBST, we added 100 µL of an alkaline phosphatase-labeled goat anti-mouse IgG secondary antibody (Thermo Fisher Scientific) diluted 1:2000 in PBST + 1% BSA for 30 min, at 37 °C. Finally, the plates were washed five times with PBST and developed with 100 µL of the 1-step PNP substrate (Thermo Fisher Scientific) for 30 min at 37 °C. The reaction was stopped by adding 100 µL of 1 M NaOH and the absorbance was read at 405 nm on a Tecan microplate reader. For the detection of α-Qβ antibodies, 96-well PolySorp plates (Thermo Fisher Scientific) were coated with 100 µL Qβ (10 µg/mL in PBS) and incubated at 4 °C overnight. After three washes in PBST, the plates were blocked with 150 µL PBST + 2% BSA at 37 °C for 1 h. The subsequent steps were as described for the detection of HPV peptides.

The IgG subtype was also determined by ELISA. The procedure was similar to that described above for Qβ and HPV peptides, but the secondary antibody was replaced with goat anti-mouse antibodies specific for IgG2a, IgG2b, and IgG1 (diluted 1:2000), as part of the IgG subtype antibody kit (Sigma-Aldrich, St. Louis, MO, USA). After three washes in PBST, we added an alkaline phosphatase-labeled rabbit anti-goat antibody (Sigma-Aldrich) diluted 1:3000, then washed five times in PBST. Finally, the plates were developed by adding the 1-step PNP substrate, as described above. For all ELISAs, the antibody titers were defined as the reciprocal serum dilution at which the absorbance exceeded the background value by >0.2.

### 2.11. Pseudovirus Production and Purification

HPV16 pseudovirus encapsidating the reporter plasmid pfwB (Addgene #37329) encoding green fluorescent protein (GFP) was produced in 293 TT cells, as previously described [30]. In brief, 293 TT cells were transfected with a mixture of two plasmids—pfwB and p16Llw (Addgene #37320) containing the sequence of each HPV shell. After 48 h, the cells were lysed and mature pseudovirus was purified on an Optiprep gradient (27:33:39) through ultracentrifugation at 125,755× *g* for 6 h. Gradient fractions corresponding to the L1 band were collected and characterized by SDS-PAGE, agarose gel electrophoresis, and flow cytometry, as recommended [30]. HPV16 pseudovirus stocks were titrated in pgsa-745 cells to yield 50–60% GFP-positive cells (percentage of infectivity) for the L2-based neutralization assay.

### 2.12. L2-Based Neutralization Assay

MCF10A cells were cultured in 96-well plates to produce the extracellular matrix (ECM), followed by the addition of the HPV pseudovirus (PsHPV), as previously described [26]. The ECM-PsHPV were incubated overnight, then removed and replaced with two-fold serial-dilutions of serum collected from immunized mice or the growth medium, as a control. The plates were incubated for 6 h before adding pgsa-745 cells. After 48 h, HPV16 pseudovirus infectivity and neutralization were assessed by flow cytometry (Accuri) based on the expression levels of GFP in infected cells. The reciprocal of the highest serum dilution that inhibited 50% of the infection relative to the control serum was considered the neutralization titer.

### 2.13. Statistical Analysis

Comparisons of the differences between two different groups were performed using unpaired two-tailed student’s *t*-test (GraphPad Prism software); * *p* < 0.05; ** *p* < 0.01; *** *p* < 0.005; ns, not significant *p* > 0.05. Values were expressed as means ± standard deviations. Sample sizes are stated in the figure legends.

## 3. Results and Discussion

**Figure 1:**
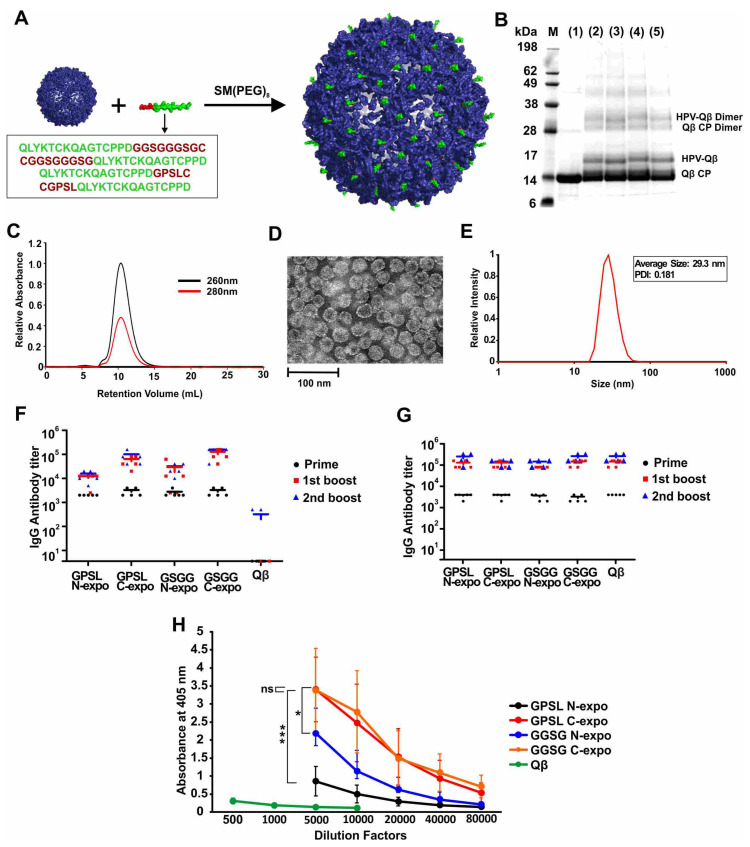
The synthesis, characterization, and immunogenicity of the HPV-Qβ conjugates. (**A**) Schematic representation of the synthesis of four HPV-Qβ conjugates differing in the linker structure and epitope orientation. (**B**) SDS-PAGE analysis of the Qβ and HPV-Qβ particles: (1) Qβ, (2) GPSL-N-expo-HPV-Qβ, (3) GPSL-C-expo-HPV-Qβ, (4) GGSG-N-expo-HPV-Qβ, and (5) GGSG-C-expo-HPV-Qβ. (**C**) FPLC chromatogram of GGSG-C-expo-HPV-Qβ. (**D**) TEM image of GGSG-C-expo-HPV-Qβ (scale bar = 100 nm). (**E**) DLS analysis of GGSG-C-expo-HPV-Qβ. (**F**) Serum IgG titers against the HPV peptide following immunization with the four HPV-Qβ conjugates or Qβ. (**G**) Serum IgG titers against Qβ following immunization with the four HPV-Qβ conjugates or Qβ. (**H**) ELISA signals at 405 nm for mice vaccinated with different antigens at different dilutions after the second boost. Mice were subcutaneously injected with 30 µg of each agent on day 0 (prime), 14 (first boost), and 28 (second boost). Blood was collected 7 days after each injection (Data are means ± S.D. for n = 5 mice per group). Asterisks show significance as determined by unpaired two-tailed student’s *t*-test: ns, not significant *p* > 0.05; * *p* < 0.05; *** *p* < 0.005.

The HPV16 epitope L2_17–31_ is ideal for the development of an effective HPV vaccine because it is highly conserved among diverse HPV isolates [15,16,17]. Different linkers and epitope orientations can have a significant effect on the immunogenicity of peptides [31,32], thus, we designed four peptides containing the L2_17–31_ epitope, featuring alternative linkers (GPSL or GGSGGGSG) and orientations (epitope exposed at the N-terminus or C-terminus), as shown in Table S1. The peptides were conjugated to the surface of the VLPs derived from bacteriophage Qβ in a two-step procedure, where a bifunctional PEG was first conjugated via the NHS-chemistry to surface amines of Qβ. Next, terminal cysteines from the HPV epitopes were conjugated via a maleimide linker to the PEG-Qβ conjugate (Figure 1A). SDS-PAGE analysis of the products confirmed the presence of unmodified Qβ coat protein with a molecular weight of 14.2 kDa and an additional band at ~17 kDa, corresponding to the coat protein conjugated to the HPV peptide (Figure 1B). Unmodified and conjugated VLPs retained the dimeric assemblies under the electrophoresis conditions, as expected due to the relative strength of the dimeric interaction. The relative intensity of the bands indicated that ~30% of the Qβ coat proteins were conjugated to HPV peptides (GPSL-N-expo 32.2%, GPSL-C-expo 31.1%, GGSG-N-expo 33.8%, GGSG-C-expo 31.8%). FPLC analysis revealed a single peak, indicating that the VLPs were monodisperse and did not form aggregates (Figure 1C and Figure S1). DLS revealed that the diameter of the VLPs was 28 ± 3 nm, which was consistent with the TEM images (Figure 1D,E and Figure S1). These results indicated that the conjugation of HPV peptides did not alter the overall size or shape of the VLP as anticipated, given the low molecular weight of the peptides.

We found that all four conjugated VLPs (but not the unmodified Qβ particles) elicited the production of anti-HPV IgG in mice, but that the higher titers were achieved when the epitope was exposed at the C-terminus (Figure 1F). All four conjugated VLPs and unmodified Qβ particles generated similar IgG titers against the Qβ carrier, as would be expected for a VLP (Figure 1G). There was no significant difference in titer when comparing the two constructs with the HPV peptide exposed at the C-terminus but at different linkers, even when using different dilution factors for the second boost (Figure 1H and Figure S2). Ultimately, we selected the construct with the C-terminal epitope and the GGSGGGSG linker for the subsequent experiments and refer to this construct hereafter as HPV-Qβ. The conjugation of HPV16 epitope L2_17–31_ to Qβ was necessary to enhance its immunogenicity, because the injection of a mixture of unmodified Qβ and the free HPV peptide elicited antibodies against Qβ but not against HPV, and no antibodies against HPV were generated by the injection of the free HPV peptide alone (Figure S3). 

**Figure 2:**
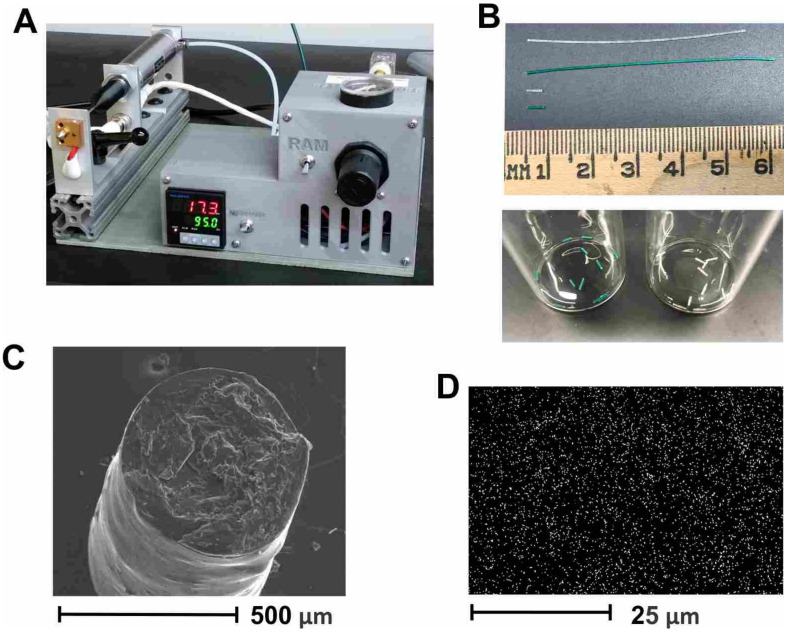
The preparation and characterization of melt-extruded PLGA implants. (**A**) Equipment for the melt processing of polymer materials. (**B**) PLGA implants produced by melt extrusion. The long implant bar produced directly from the device is shown at the top, with the short sections ready for implantation shown underneath. The photograph shows a metric ruler. The white bars are plain PLGA + Qβ-HPV, and the green ones are FITC-PLGA + Cy5-Qβ. (**C**) SEM image showing a cross-section of the PLGA implant loaded with Qβ-HPV. (**D**) The EDX spectrum sulfur K-series emission signal (SK series) map of the PLGA implant loaded with Qβ-HPV.

HPV-Qβ was encapsulated into the PLGA implants through melt-extrusion, as previously reported [25]. Parameters that were previously optimized included temperature, such that the temperature was well above the T_g_ of PLGA, to allow for flow at a reasonably low pressure and residence time in the extruder, to ensure complete melting of the polymer. This method can be easily adapted to other vaccine candidates but requires optimization for each candidate to determine temperature stability, candidate release rate, etc. To optimize the control of the implant parameters, we used a benchtop melt-processing system (Figure 2A) [29]. Taking advantage of the stable extrusion pressure and adjustable nozzle size, the PLGA implants were produced with an accurate diameter of less than 0.8 mm, so that they fit inside 18–gauge needles, for convenient administration. The loaded PLGA implants were extruded as implant cylinders, 5–6 mm in length, which were cut into segments of 0.3–1 mm for implantation (Figure 2B). SEM imaging of implant cross-sections revealed a uniform cylinder of ~0.5 mm diameter (Figure 2C). Homogeneous dispersion of HPV-Qβ particles was verified by the EDX analysis, specifically the sulfur K-series emission signal map (Figure 2D). Our prior experience indicates that homogeneous dispersion within the implant improves the linearity of release and negates the aggregation of VLPs from the released fraction.

**Figure 3:**
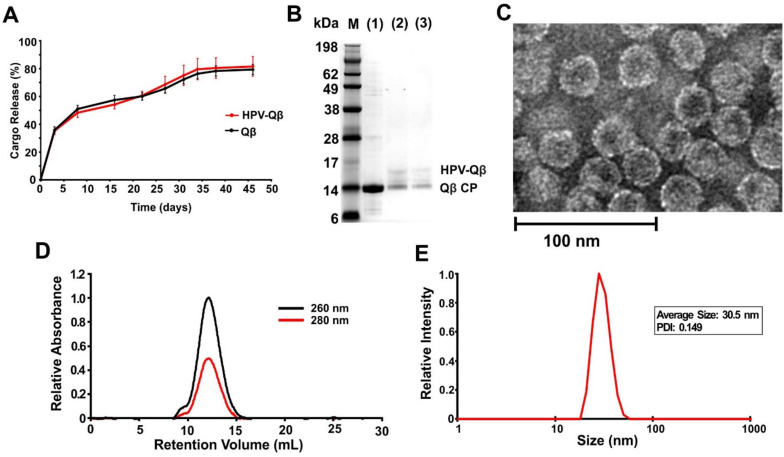
Slow release of Qβ-HPV from the PLGA implant. (**A**) The release curve of the PLGA implants loaded with Qβ-HPV or Qβ, with the particles released into PBS at 37 °C (data are means ± standard deviations, n = 3). (**B**) SDS-PAGE analysis of (1) unmodified Qβ standard and Qβ-HPV released from the implant between (2) 0–15 days and (3) 16–30 days. (**C**) TEM image of the released Qβ-HPV particles on day 30. (**D**) FPLC analysis of the released Qβ-HPV particles on day 30. (**E**) DLS analysis of the released Qβ-HPV particles on day 30.

The loaded PLGA implants were shown to slowly release the VLPs into PBS, with continuous release occurring over a period of up to 35 days at 37 °C (Figure 3A). Released fractions indicated that the HPV peptides remained conjugated following the melt-extrusion process, as confirmed by the presence of ~17 kDa bands when the samples taken on days 15 and 30 were analyzed by SDS-PAGE (Figure 3B). TEM and DLS confirmed the presence of intact HPV-Qβ particles with a diameter of ~30 nm (Figure 3C,D). FPLC analysis of the released HPV-Qβ particles showed that the melt-extrusion process did not promote aggregation either during encapsulation or upon release (Figure 3E).

The in vivo subcutaneous environment is more complex than PBS and might influence the release of the cargo from the PLGA implants. We sought to evaluate the release kinetics of the implanted Qβ using fluorescence molecular tomography in vivo. Unmodified Qβ was used for this set of studies, rather than HPV-Qβ due to the competing chemical reactions for surface conjugation of fluorophores (i.e., HPV-Qβ exhausted the most reactive amines for conjugation). To track the release of the particles in vivo, Qβ was labeled with Cy5 and the incorporation of the fluorophore was confirmed by FPLC and SDS-PAGE (Figure S4). FPLC analysis revealed a peak in the 647 nm channel (Cy5 absorbance), at a retention volume of ~11 mL, corresponding to the Qβ particles (Figure S4A). SDS-PAGE analysis showed a band corresponding to the anticipated molecular weight of Cy5-Qβ, with overlapping signals in the blue fluorescence and Coomassie Brilliant Blue channels (Figure S4B).

**Figure 4:**
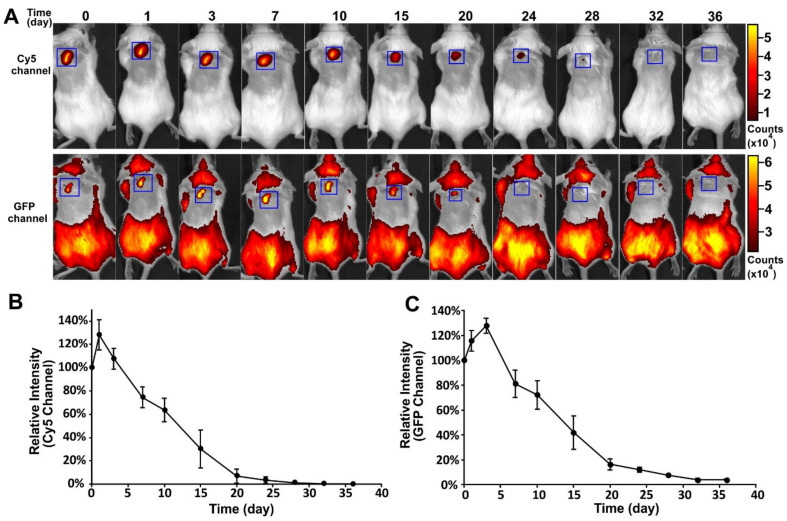
Release of Cy5-Qβ particles from PLGA implants in vivo. (**A**) Fluorescence images of mice containing FITC-PLGA implants loaded with Cy5-Qβ, showing the Cy5 channel (top) and GFP channel (bottom) at different time-points. Blue square shows implant location. Scale bar shows fluorescence intensity for all images in each channel. (**B**) Quantitative release profile (Cy5 channel) for the FITC-PLGA implants loaded with Cy5-Qβ (Data are means ± s.d. for n = 4 mice per group). (**C**) Quantitative release profile (GFP channel) for the FITC-PLGA implants loaded with Cy5-Qβ (Data are means ± s.d. for n = 4 mice per group).

To track the in vivo fate of the encapsulated VLPs and the polymeric implant, an FITC-PLGA matrix loaded with Cy5-Qβ particles was implanted subcutaneously into mice and fluorescence images were captured at different time-points (Figure 4A). This allowed for separate imaging of polymer degradation and cargo release. The profiles of the Cy5 and GFP channels are shown in Figure 4B,C, respectively. A slight increase in fluorescence intensity was observed in both channels, immediately after implantation. The fluorescence intensity in both channels then declined continually from day 3 to day 32, indicating the degradation of the PLGA and the slow release of the VLPs. The degradation of PLGA was closely correlated to the decline of the VLP signal. This experiment confirmed that the PLGA implants produced by melt-extrusion retained their ability to release cargo slowly over time in vivo and that degradation kinetics of the polymer play the main role in cargo release.

**Figure 5:**
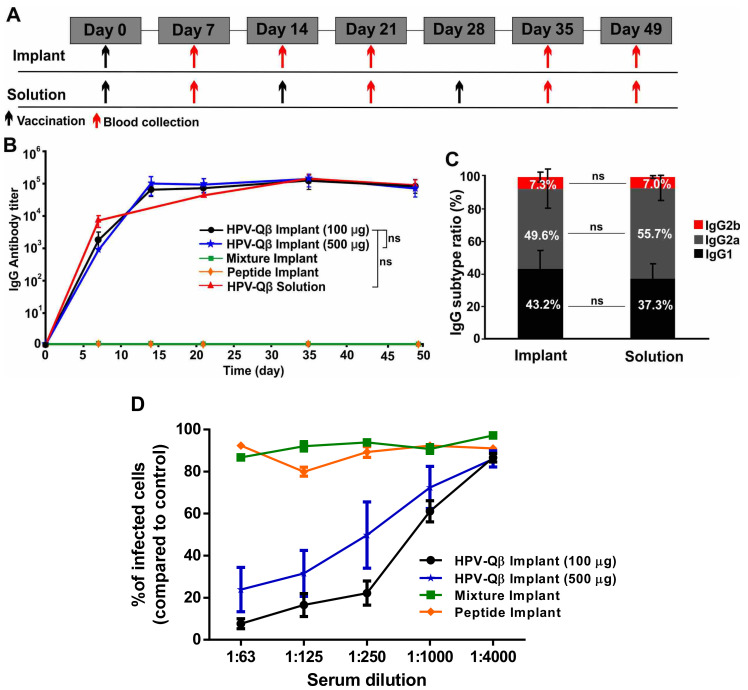
Single-dose vaccination using PLGA implants loaded with HPV-Qβ. (**A**) Vaccination and bleeding schedule for mice with subcutaneous PLGA implants or equivalent HPV-Qβ injections. (**B**) Serum titers of HPV-specific IgG for mice vaccinated with three subcutaneous injections of 30 µg HPV-Qβ or a single-dose PLGA implant loaded with 100 µg HPV-Qβ, 500 µg HPV-Qβ, a mixture of 100 µg Qβ and 20 µg HPV peptide, or 20 µg HPV peptide alone (Data are means ± s.d. for n = 6 mice per group). Statistical significance was determined by unpaired two-tailed student’s *t*-test: ns, not significant with *p* > 0.05. (**C**) HPV-specific IgG subtype ratio for mice vaccinated with a single-dose PLGA implant containing 100 µg HPV-Qβ, or three subcutaneous doses of 35 µg HPV-Qβ. Blood was collected on day 35 (Data are means ± s.d. for n = 6 mice per group). (**D**) Serum (day 35) from three mice immunized with various vaccine formulations tested in duplicate for neutralization against HPV16 pseudovirus at ID_60_ (pseudovirus infectious dose that infects 60–70% of control cells). Infected cells (expressing GFP) were identified by flow cytometry (data are means ± standard errors based on the relative percentage of infected cells in wells exposed to serum compared to non-exposed controls).

To assess the efficacy of the vaccine candidate delivered using the vaccine delivery device, we subcutaneously vaccinated the mice with PLGA implants loaded with HPV-Qβ at two different doses (100 µg and 500 µg). As controls, we vaccinated mice with PLGA implants loaded with a mixture of the HPV peptide and unmodified Qβ, or with the HPV peptide alone. Finally, we also subcutaneously immunized mice with three doses of free HPV-Qβ (30 µg per dose), as a conventional vaccination control. The immunization and bleeding schedule is summarized in Figure 5A.

We compared the anti-HPV IgG titers in serum samples taken from mice in each of the experimental and control groups (Figure 5B). The PLGA implants loaded with HPV-Qβ elicited an intense immune response, with the high HPV-specific IgG titers starting from day 14 and lasting to day 49. There was no significant difference in the IgG titers between the groups receiving 100 µg and 500 µg HPV-Qβ in the implant, indicating that 100 µg HPV-Qβ is an adequate quantity of the antigen for immunization. The high titers elicited by the single-dose HPV vaccine implant were maintained over 49 days and were similar to the titers elicited by the prime, first boost, and second boost schedule, with free VLPs. The PLGA implants loaded with the HPV peptide or a mixture of the HPV peptide and unmodified Qβ particles did not elicit a significant immune response against HPV, demonstrating that the PLGA matrix had no impact on the immunogenicity of the antigens but only ensured their slow release (Figure 5B). The anti-Qβ IgG titer was consistent with that against the HPV peptide. High IgG titers against Qβ were detected from day 14 to day 49 in mice vaccinated with PLGA implants containing HPV-Qβ or the mixture of unmodified Qβ and the HPV peptide (Figure S5A). 

The IgG subtype ratio following vaccination indicates the mechanism of the immune response. Mice vaccinated with the single-dose HPV-Qβ vaccine based on the PLGA implant, showed a similar HPV-specific IgG subtype ratio to mice in the conventional vaccination control group (Figure 5C). In both cases, IgG2a was the predominant subtype (~50%), followed by IgG1 (~40%) and IgG2b (~7%). Interestingly, the Qβ-specific IgG subtype ratio was distinct, with IgG2a accounting for up to 70% and IgG1 only 15% of the total IgG titer in both single-dose vaccine group and the conventional vaccination control group (Figure S5B). This indicates that the cleavage of the bond between the HPV epitope and Qβ carrier is a specific step during immune response, with the separate components then following different immune response pathways. 

Additionally, we aimed to evaluate to compare a single-dose soluble injection versus a single-dose biodegradable implant, in an effort to evaluate the necessity for a prime-boost regime with this vaccine candidate. Three groups were studied—(1) a single dose of biodegradable 100 μg HPV-Qβ loaded PLGA implant, (2) a single dose of subcutaneous injection of 100 μg of HPV-Qβ solution, and (3) three subcutaneous injections of 30 µg HPV-Qβ solution. The vaccination and bleeding schedule are shown in Figure S6A. Data indicate comparable levels of HPV-specific IgG antibody titers independent of the schedule (prime boost vs. single administration) or delivery strategy (injection vs. implant), throughout the entire time of observation for up to 90 days (Figure S6B,C). This indicates that this particular HPV-Qβ vaccine candidate might be effective after single dosing.

We find that in the case of slow release from the implant, the IgG antibody titer was slightly lower after 14 days and reached the same level as obtained by the other methods after 28 days. In all cases, the antibodies remained similar even after three months. Since the lifetime of HPV antibodies in mouse is found to be much longer (>90 days) than the release time of the vaccine cargo from the implant (about 20–25 days), a simple model of exponential loss of antibody in the mouse and an approximately linear release of vaccine cargo from the implant supports our observations (see Appendix S1). The model demonstrates that decay kinetics would dominate the concentration of antibodies in blood in the long run and would be essentially same for all methods of administration for this vaccine. Hence, we conclude that the mice vaccinated with the slow-release implants obtain, after a reasonable time, IgG titers comparable to that obtained by the traditional one single full dose or three-dose strategy. While this result might be disappointing, we would like to point out that subcutaneous injections are very expensive and require trained healthcare professionals, thus limiting its availability in developing and poor countries. Furthermore, we speculate that at a lower dose, the implant group might show some advantages of the single dose injection; and this will be tested in future work. If this holds true; this would reduce production costs, which would also be of benefit. Nevertheless, the implications of melt manufacturing of implantable devices has the potential to be immense. Microneedle patches can be manufactured through injection molding, which has the advantage of massive scalability. These devices show similar efficacy when self-administered, and given the known stability of Qβ, might eliminate cold-chain requirements. We anticipate that this technique would be of great value for vaccines that require a prime-boost dosing regimen. Additionally, several recent studies also point out the benefits of sustained release of antigen over bolus injection in terms of better antigen recognition features and enhancement of germinal center responses. [33,34].

In order to demonstrate the efficacy of the single-dose HPV-Qβ vaccine, serum antibodies from the vaccinated mice were tested for their ability to prevent the infection of pgsa-745 cells by HPV pseudovirus in an in vitro neutralization assay (Figure S7), as previously reported for other vaccine candidates [26]. Data indicate that antibodies raised were indeed neutralizing. The relative number of infected GFP-positive cells was significantly lower when the serum from animals immunized with HPV-Qβ (100 or 500 µg) was added during the assay, compared to serum from animals vaccinated with a mixture of unmodified Qβ and the HPV peptide, or the HPV peptide alone (Figure 5D). The neutralization titers for each treatment were 1:750 for HPV-Qβ (100 µg), 1:250 for HPV-Qβ (500 µg), and effectively zero neutralizing activity for the free peptide, with or without unmodified Qβ.

## 4. Conclusions

Subunit vaccine candidates were successfully formulated using conserved L2 peptide epitopes and the bacteriophage Qβ display nanotechnology. The HPV-Qβ were more immunogenic if the HPV peptides were exposed at the C-terminus rather than the N-terminus, and the best-performing VLP elicited a strong immune response in mice. The HPV-Qβ conjugate was encapsulated into PLGA implants through melt-extrusion, which can be controlled precisely using a new benchtop melt-processing system that recapitulates large-scale polymer manufacturing platforms. The immunogenicity of HPV-Qβ conjugate was retained during this process. The PLGA implants released the VLP cargo slowly, both in vitro and in vivo. Most importantly, the titer of IgG against the HPV peptide was similar in mice receiving a single-dose vaccination of HPV-Qβ via the PLGA implant and in those receiving traditional soluble vaccinations of HPV-Qβ. Furthermore, serum antibodies from mice vaccinated with the implant were able to block the infection of cells by HPV pseudovirus in an in vitro neutralization assay. Our results therefore demonstrate the feasibility and efficacy of a single-dose HPV vaccine based on a biodegradable polymer implant. This is of high importance for future development of self-administered vaccine devices made by polymer melt processing. It is also important to note that the high temperature stability of the HPV-Qβ vaccine candidate serves two functions—(1) it allows for traditional polymer manufacturing methods to scale-up fabrication of the implant, and (2) the vaccine and its implant would eliminate the need for the cold chain, thus enabling global distribution. The design concepts are highly adaptable and would be applicable to any infectious disease. The concepts described are especially appealing because they are scalable and fit within the traditional workflow of pharmaceutical production. Bacterial fermentation is a common method to generate therapeutic proteins and VLPs provide an especially high yielding platform with minimal optimization (Qβ evolved to be expressed in bacteria). Likewise, the device platform is common in the pharmaceutical industry, where hot melt extrusion is a common method to make pills and devices. The vaccine administration described herein mimics that of the clinically approved device, Nexplanon. Finally, since the VLP serves as a carrier, new epitopes can be quickly adapted to new diseases or strains.

## Data Availability

Data available upon request.

## Supplementary Materials

The following are available online at https://www.mdpi.com/2076-393X/9/1/66/s1. Figure S1: Characterization of Qβ and HPV-Qβ conjugates by TEM, FPLC, and DLS. (A) Qβ, (B) GPSL-N-expo-HPV-Qβ, (C) GPSL-C-expo-HPV-Qβ, and (D) GGSG-N-expo-HPV-Qβ. Figure S2: ELISA signals at 405 nm for mice vaccinated with various antigens at different dilutions after (A) prime vaccination and (B) the first boost (Data are means ± s.d. for n = 5 mice per group). Asterisks show significance, as determined by unpaired two-tailed student’s *t*-test: ns, not significant *p* > 0.05; * *p* < 0.05; ** *p* < 0.01; *** *p* < 0.005. Figure S3: Vaccination with HPV-Qβ solutions. The serum IgG antibody titer against (A) the HPV peptide and (B) Qβ for mice vaccinated with 30 µg HPV-Qβ, a mixture of 5 µg HPV and 30 µg Qβ, or 5 µg HPV peptide alone. Mice were subcutaneously injected with the agents on days 0 (prime), 14 (first boost), and 21 (second boost). Blood was collected 7 days after each injection (n = 6 for each group). Figure S4: The characterization of Cy5-Qβ. (A) FPLC chromatogram of Cy5-Qβ. (B) SDS-PAGE analysis of Cy5-Qβ (lane 1) and unmodified Qβ (lane 2). The Coomassie Brilliant Blue channel is shown on the left, and the blue fluorescence channel on the right; M = size markers. Figure S5: Immunization of mice with Qβ particles. (A) The serum titer of Qβ-specific IgG in mice vaccinated with three subcutaneous injections of 30 µg HPV-Qβ or a single dose of 100 µg HPV-Qβ, 500 µg HPV-Qβ, a mixture of 100 µg Qβ and 20 µg HPV peptide, or 20 µg HPV peptide alone, in each case loaded on a subcutaneous PLGA implant. (B) Qβ-specific IgG subtype ratio in mice vaccinated with a single-dose PLGA implant containing 100 µg HPV-Qβ, or three doses of 35 µg HPV-Qβ (Data are means ± s.d. for n = 6 mice per group). Statistical significance was determined by unpaired two-tailed student’s *t*-test; ns, not significant with *p* > 0.05. Figure S6: Immunization with HPV-Qβ at different doses and schedules. Mice were immunized with (i) single dose PLGA implant loaded with 100 µg HPV-Qβ (ii) single subcutaneous injection of 100 μg HPV-Qβ solution, and (iii) three subcutaneous injections of 30 µg HPV-Qβ solution. Six mice were studied per group. (A) Schedule for vaccination (black arrow) and bleeding (red arrow) for all three vaccine formulations. Blood collection was done every two weeks. (B) Serum titers of HPV-specific IgG for vaccinated mice on day 14 (black), day 28 (red), day 42 (blue), day 56 (cyan), and day 90 (orange). The scattered points represent individual mouse and the horizontal lines represent the mean for each group. (C) Kinetic profile of HPV-specific IgG antibody titer for mice groups vaccinated with different vaccine formulation—100 µg of HPV-Qβ loaded implant (black), single subcutaneous injection of 100 µg HPV-Qβ solution (blue), and three subcutaneous injections of 30 µg HPV-Qβ solution (red). Error bars represent uncertainties (±1σ) for each mice group.Figure S7. Production and characterization of HPV16 pseudovirus (PsHPV). (A) Pseudovirus purification after ultracentrifugation. The red arrow indicates the band corresponding to the intact pseudovirus particles. (B) Different fractions (1–18) of pseudovirus particles prepared by ultracentrifugation and separated by 2% agarose gel electrophoresis. The presence of bands indicates pseudovirus DNA. (C) SDS-PAGE analysis (14–20% gels) of the same fractions tested in B, showing resolved bands representing L1 (50–55 kDa) and histone (15 kDa). Fractions 9–18 were considered pseudovirus positive and were used for the neutralization assay. MW = molecular weight. (D) Pooled fractions (9–18) titrated and analyzed by flow cytometry. The presence of GFP-positive cells indicates an infection.

## References

[1] Centers for Disease Control and Prevention (2014). Genital HPV Infection—CDC Fact Sheet.

[2] Zhai L., Tumban E. (2016). Gardasil-9: A global survey of projected efficacy. Antivir. Res..

[3] Petrosky E., Bocchini J.A., Hariri S., Chesson H., Curtis C.R., Saraiya M., Unger E.R., Markowitz L.E. (2015). Use of 9-valent human papillomavirus (HPV) vaccine: Updated HPV vaccination recommendations of the advisory committee on immunization practices. Mmwr. Morb. Mortal. Wkly. Rep..

[4] Centers for Disease Control and Prevention (2010). FDA licensure of bivalent human papillomavirus vaccine (HPV2, Cervarix) for use in females and updated HPV vaccination recommendations from the Advisory Committee on Immunization Practices (ACIP). Mmwr. Morb. Mortal. Wkly. Rep..

[5] Centers for Disease Control and Prevention (2012). HPV Vaccine Information for Clinicians—Fact Sheet.

[6] Kirby T. (2015). FDA approves new upgraded Gardasil 9. Lancet Oncol..

[7] Walker T.Y., Elam-Evans L.D., Yankey D., Markowitz L.E., Williams C.L., Fredua B., Singleton J.A., Stokley S. (2019). National, regional, state, and selected local area vaccination coverage among adolescents aged 13–17 years—United States, 2018. Morb. Mortal. Wkly. Rep..

[8] Stokley S., Jeyarajah J., Yankey D., Cano M., Gee J., Roark J., Curtis C.R., Markowitz L. (2014). Human papillomavirus vaccination coverage among adolescents, 2007–2013, and postlicensure vaccine safety monitoring, 2006–2014—United States. Mmwr. Morb. Mortal. Wkly. Rep..

[9] Wigle J., Fontenot H.B., Zimet G.D. (2016). Global Delivery of Human Papillomavirus VACCINES Human Papillomavirus HPV Vaccine Immunization Global Progress. Pediatr. Clin. N. Am..

[10] Reagan-Steiner S., Yankey D., Jeyarajah J., Elam-Evans L.D., Singleton J.A., Curtis C.R., MacNeil J., Markowitz L.E., Stokley S. (2015). National, regional, state, and selected local area vaccination coverage among adolescents aged 13–17 years—United States, 2014. Mmwr. Morb. Mortal. Wkly. Rep..

[11] Trus B., Buck C., Cheng N., Lowy D., Steven A., Schiller J. (2005). Localization of the HPV-16 minor capsid protein L2 by difference imaging. Microsc. Microanal..

[12] Buck C.B., Cheng N., Thompson C.D., Lowy D.R., Steven A.C., Schiller J.T., Trus B.L. (2008). Arrangement of L2 within the papillomavirus capsid. J. Virol..

[13] Chen X.S., Garcea R.L., Goldberg I., Casini G., Harrison S.C. (2000). Structure of small virus-like particles assembled from the L1 protein of human papillomavirus 16. Mol. Cell.

[14] European Medicines Agency (2015). Gardasil 9 Public Assessment Report.

[15] Tyler M., Tumban E., Dziduszko A., Ozbun M.A., Peabody D.S., Chackerian B. (2014). Immunization with a consensus epitope from human papillomavirus L2 induces antibodies that are broadly neutralizing. Vaccine.

[16] Tumban E., Peabody J., Peabody D.S., Chackerian B. (2011). A pan-HPV vaccine based on bacteriophage PP7 VLPs displaying broadly cross-neutralizing epitopes from the HPV minor capsid protein, L2. PLoS ONE.

[17] Zhai L., Peabody J., Pang Y.-Y.S., Schiller J., Chackerian B., Tumban E. (2017). A novel candidate HPV vaccine: MS2 phage VLP displaying a tandem HPV L2 peptide offers similar protection in mice to Gardasil-9. Antivir. Res..

[18] Shao S., Geng J., Yi H.A., Gogia S., Neelamegham S., Jacobs A., Lovell J.F. (2015). Functionalization of cobalt porphyrin–phospholipid bilayers with his-tagged ligands and antigens. Nat. Chem..

[19] Shukla S., Myers J.T., Woods S.E., Gong X., Czapar A.E., Commandeur U., Huang A.Y., Levine A.D., Steinmetz N.F. (2017). Plant viral nanoparticles-based HER2 vaccine: Immune response influenced by differential transport, localization and cellular interactions of particulate carriers. Biomaterials.

[20] Xia Y., Wu J., Wei W., Du Y., Wan T., Ma X., An W., Guo A., Miao C., Yue H. (2018). Exploiting the pliability and lateral mobility of Pickering emulsion for enhanced vaccination. Nat. Mater..

[21] Yue H., Ma G. (2015). Polymeric micro/nanoparticles: Particle design and potential vaccine delivery applications. Vaccine.

[22] Demento S.L., Cui W., Criscione J.M., Stern E., Tulipan J., Kaech S.M., Fahmy T.M. (2012). Role of sustained antigen release from nanoparticle vaccines in shaping the T cell memory phenotype. Biomaterials.

[23] Desai K.G.H., Schwendeman S.P. (2013). Active self-healing encapsulation of vaccine antigens in PLGA microspheres. J. Control. Release Off. J. Control. Release Soc..

[24] Irvine D.J., Aung A., Silva M. (2020). Controlling timing and location in vaccines. Adv. Drug Deliv. Rev..

[25] Lee P.W., Shukla S., Wallat J.D., Danda C., Steinmetz N.F., Maia J., Pokorski J.K. (2017). Biodegradable viral nanoparticle/polymer implants prepared via melt-processing. ACS Nano.

[26] Mohsen M.O., Gomes A.C., Cabral-Miranda G., Krueger C.C., Leoratti F.M., Stein J.V., Bachmann M.F. (2017). Delivering adjuvants and antigens in separate nanoparticles eliminates the need of physical linkage for effective vaccination. J. Control. Release.

[27] Bao Q., Li X., Han G., Zhu Y., Mao C., Yang M. (2019). Phage-based vaccines. Adv. Drug Deliv. Rev..

[28] Isarov S.A., Lee P.W., Pokorski J.K. (2016). “Graft-to” Protein/Polymer Conjugates Using Polynorbornene Block Copolymers. Biomacromolecules.

[29] Wirth D.M., Pokorski J.K. (2019). Design and fabrication of a low-cost pilot-scale melt-processing system. Polymer.

[30] Day P.M., Pang Y.-Y.S., Kines R.C., Thompson C.D., Lowy D.R., Schiller J.T. (2012). A human papillomavirus (HPV) in vitro neutralization assay that recapitulates the in vitro process of infection provides a sensitive measure of HPV L2 infection-inhibiting antibodies. Clin. Vaccine Immunol..

[31] Ahlborg N., Nardin E.H., Perlmann P., Berzins K., Andersson R. (1998). Immunogenicity of chimeric multiple antigen peptides based on Plasmodium falciparum antigens: Impact of epitope orientation. Vaccine.

[32] Monso M., De La Torre B., Blanco E., Moreno N., Andreu D. (2013). Influence of conjugation chemistry and B epitope orientation on the immune response of branched peptide antigens. Bioconjug. Chem..

[33] Cirelli K.M., Crotty S. (2017). Germinal center enhancement by extended antigen availability. Curr. Opin. Immunol..

[34] Tam H.H., Melo M.B., Kang M., Pelet J.M., Ruda V.M., Foley M.H., Hu J.K., Kumari S., Crampton J., Baldeon A.D. (2016). Sustained antigen availability during germinal center initiation enhances antibody responses to vaccination. Proc. Natl. Acad. Sci. USA.

