# Supplementary material for: A Scalable Manufacturing Approach to Single Dose Vaccination against HPV
Source: Vaccines (Basel). 2021 Jan 19;9(1):66. doi: 10.3390/vaccines9010066 (PMC7835769; doi:10.3390/vaccines9010066)
Supplement: Supplementary file 1 [file vaccines-09-00066-s001.pdf]

Supporting information

Name	Sequence	Mass (expected/observed)	Purity(%)
GPSL N-expo	QLYKTCKQAGTCPPDGPSLC	2110.5/2110.2	96.5
GPSL C-expo	CGPSLQLYKTCKQAGTCPPD	2110.5/2110.5	97.3
GGSG N-expo	QLYKTCKQAGTCPPDGGSGGGSGC	2272.5/2272.3	96.4
GGSG C-expo	CGGSGGGSGQLYKTCKQAGTCPPD	2272.5/2272.2	95.3

Table S1. Properties of the HPV peptides.

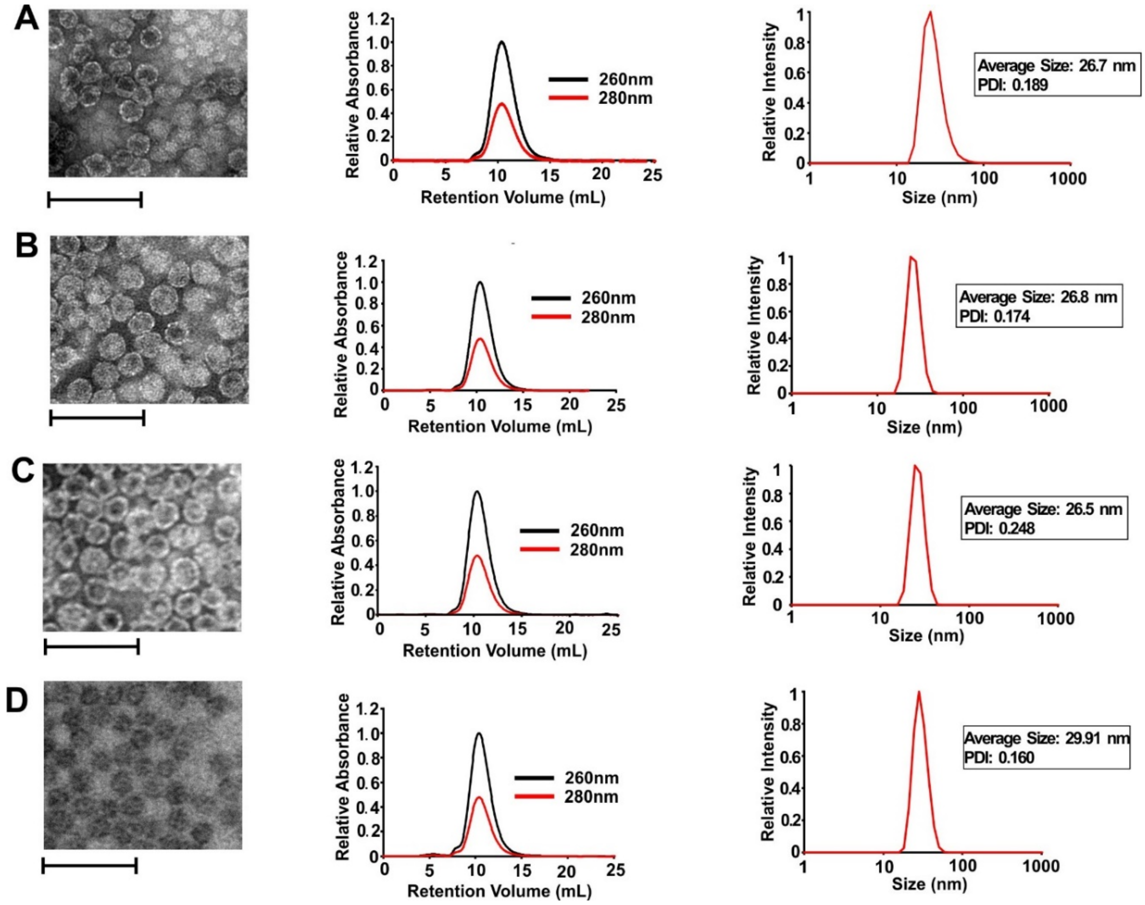

Figure S1. Characterization of Qβ and HPV-Qβ conjugates by TEM, FPLC and DLS. A) Qβ, B) GPSL-N-expo-HPV-Qβ, C) GPSL-C-expo-HPV-Qβ, and D) GGSG-N-expo-HPV-Qβ.

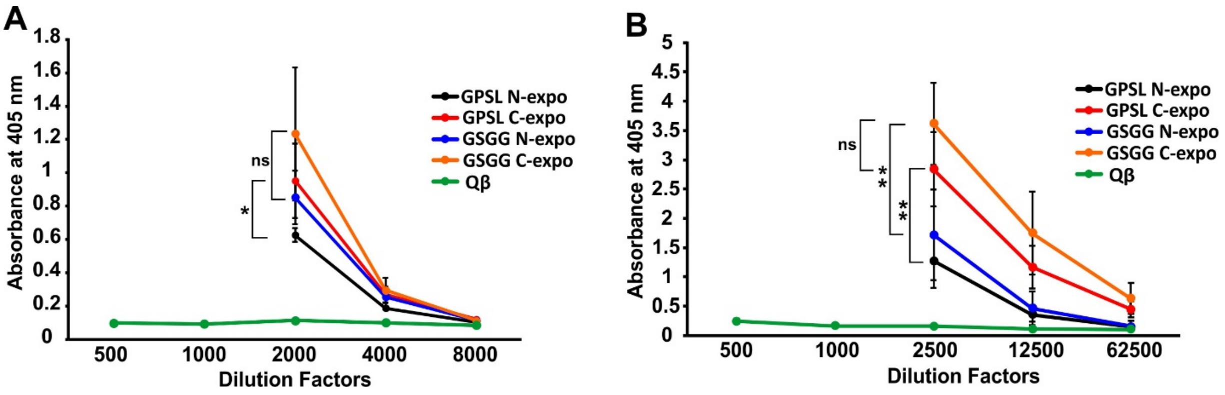

Figure S2. ELISA signals at 405 nm for mice vaccinated with various antigens at different dilutions after A) prime vaccination and B) the first boost (Data are means ± s.d. for n = 5 mice per group). Asterisks show significance as determined by unpaired two-tailed student's t test: ns, not significant p>0.05; * p<0.05; **p<0.01; ***p<0.005.

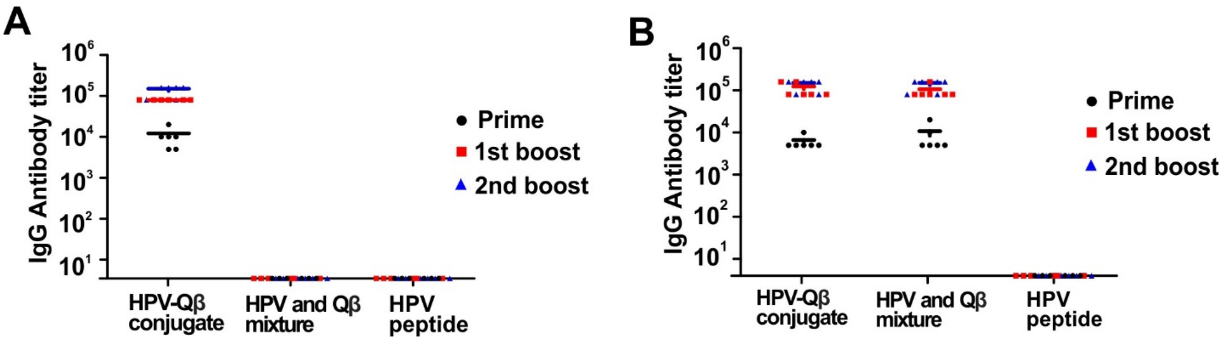

Figure S3. Vaccination with HPV-Qβ solutions. The serum IgG antibody titer against A) the HPV peptide and B) Qβ for mice vaccinated with 30 µg HPV-Qβ, a mixture of 5 µg HPV and 30 µg Qβ, or 5 µg HPV peptide alone. Mice were subcutaneously injected with the agents on days 0 (prime),

14 (first boost) and 21 (second boost). Blood was collected 7 days after each injection (n = 6 for each group).

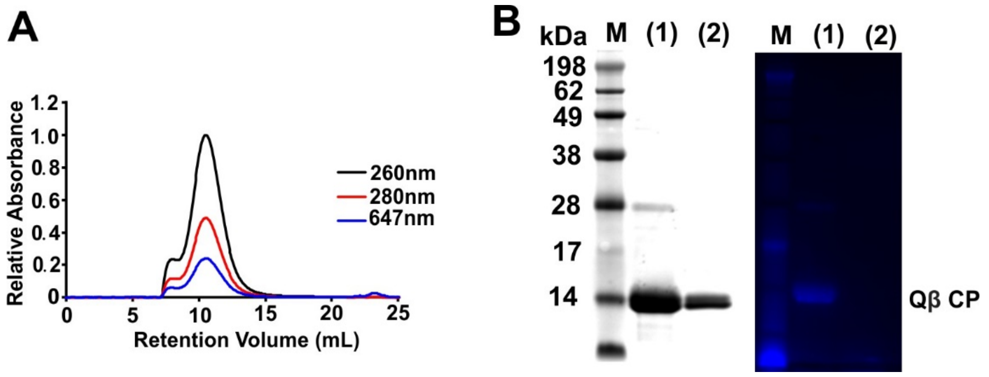

Figure S4. The characterization of Cy5-Qβ. A) FPLC chromatogram of Cy5-Qβ. B) SDS-PAGE analysis of Cy5-Qβ (lane 1) and unmodified Qβ (lane 2). The Coomassie Brilliant Blue channel is shown on the left, and the blue fluorescence channel on the right. M = size markers.

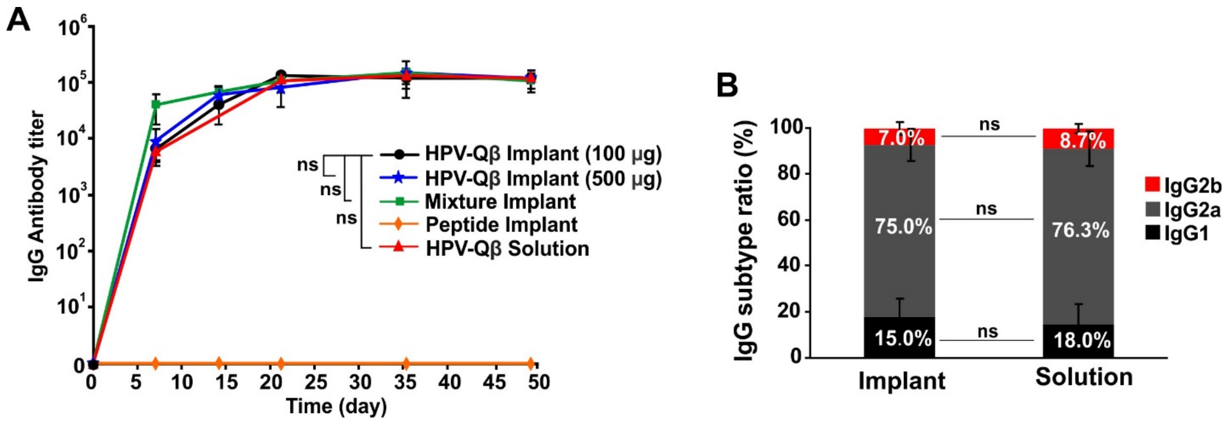

Figure S5. Immunization of mice with Qβ particles. A) The serum titer of Qβ-specific IgG in mice vaccinated with three subcutaneous injections of 30 μg HPV-Qβ or a single dose of 100 μg HPV-Qβ, 500 μg HPV-Qβ, a mixture of 100 μg Qβ and 20 μg HPV peptide, or 20 μg HPV peptide

alone, in each case loaded on a subcutaneous PLGA implant. B) Qβ-specific IgG subtype ratio in mice vaccinated with a single-dose PLGA implant containing 100 µg HPV-Qβ, or three doses of 35 µg HPV-Qβ. (Data are means ± s.d. for n = 6 mice per group). Statistical significance was determined by unpaired two-tailed student's t test: ns, not significant with p>0.05.

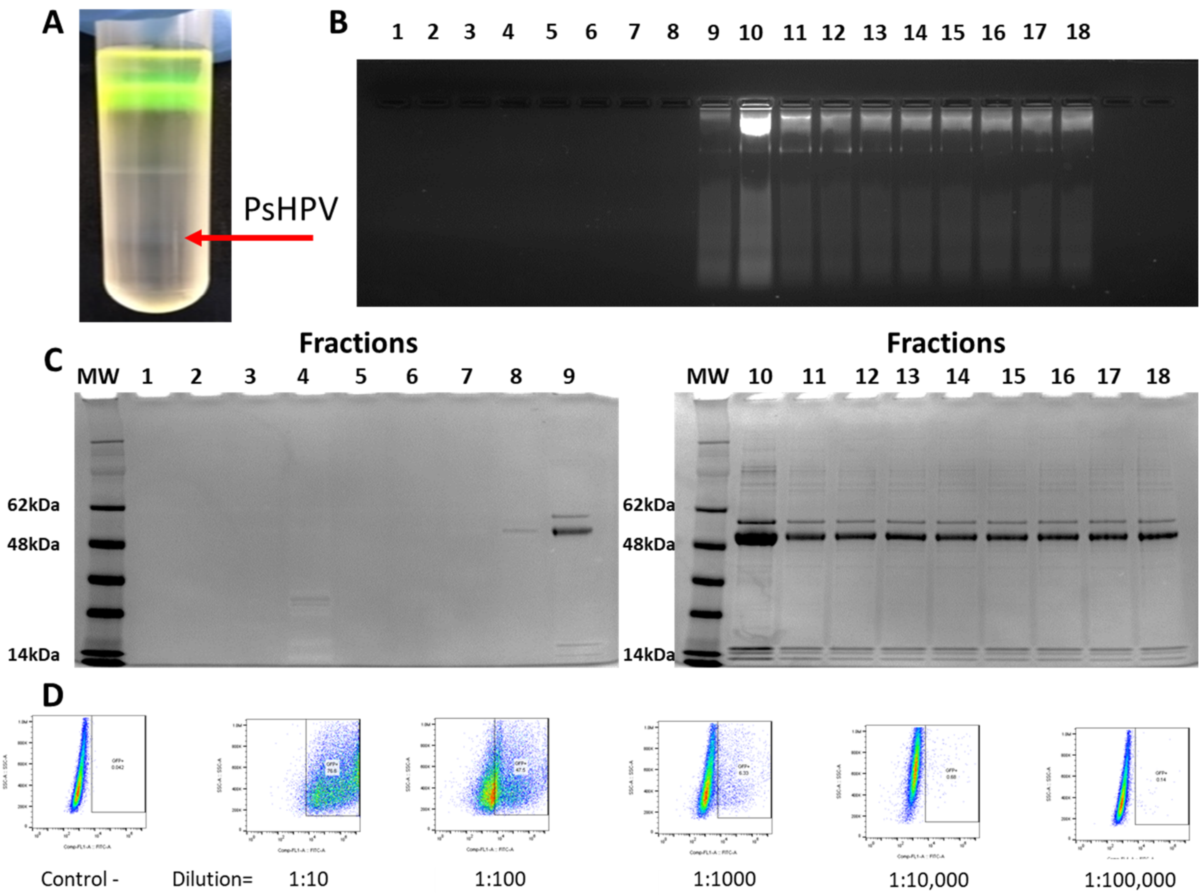

Figure S6. Production and characterization of HPV16 pseudovirus (PsHPV). A) Pseudovirus purification after ultracentrifugation. The red arrow indicates the band corresponding to the intact pseudovirus particles. B) Different fractions (1–18) of pseudovirus particles prepared by ultracentrifugation and separated by 2% agarose gel electrophoresis. The presence of bands

indicates pseudovirus DNA. C) SDS-PAGE analysis (14–20% gels) of the same fractions tested in B, showing resolved bands representing L1 (50–55 kDa) and histone (15 kDa). Fractions 9–18 were considered pseudovirus positive and were used for the neutralization assay. MW = molecular weight. D) Pooled fractions (9–18) titrated and analyzed by flow cytometry. The presence of GFP-positive cells indicates an infection.

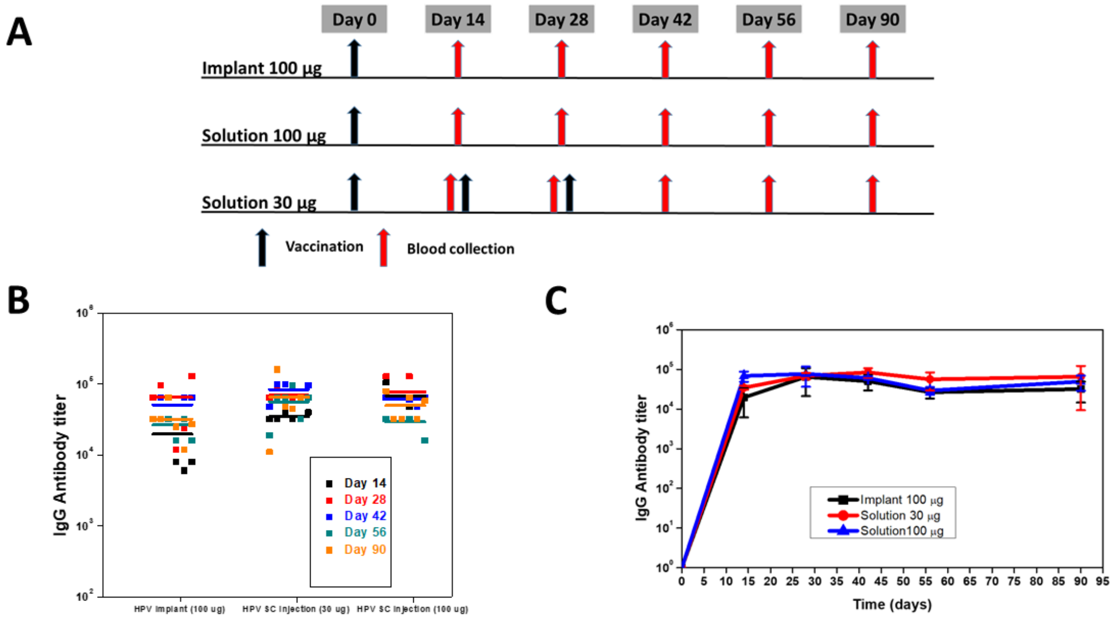

Figure S7. Immunization with HPV-Qβ at different doses and schedules. Mice were immunized with i) single dose PLGA implant loaded with 100 µg HPV-Qβ ii) single subcutaneous injection of 100 µg HPV-Qβ solution, and iii) three subcutaneous injections of 30 µg HPV-Qβ solution. Six mice were studied per group. A) Schedule for vaccination (black arrow) and bleeding (red arrow) for all three vaccine formulations. Blood collection was done every two weeks. B) Serum titers of HPV-specific IgG for vaccinated mice on day 14 (black), day 28 (red), day 42 (blue), day 56 (cyan), day 90 (orange). Scattered points represent individual mouse and horizontal lines represent

the mean for each group. C) Kinetic profile of HPV-specific IgG antibody titer for mice groups vaccinated with different vaccine formulation: 100 μg of HPV-Qβ loaded implant (black), single subcutaneous injection of 100 μg HPV-Qβ solution (blue), three subcutaneous injections of 30 μg HPV-Qβ solution (red). Error bars represent uncertainties (±1σ) for each mice group.

Appendix S1. Exponential Decay Kinetics, Produced from Slow Release Implant

Broadly, decay of antibody in a mouse follows the well-known exponential decay law. Let λ be the decay constant (lifetime $=\frac{1}{\lambda}$) of the HPV antibodies in mice. For single dose subcutaneous injection, N_0 number of HPV antibodies were produced in a mouse at once (say, time T=0). Then after a time T, the number of antibodies present in the mouse is

$N_1(T) = N_0 e^{-\lambda T}$ ....(1)

Now consider a slow release process. Let an implant be inserted in the mouse and N_0 number of antibodies were produced within the mouse in a total time-period of T_R in n number of discrete steps. Assuming a linear release as observed from **Figure 4** in the manuscript, following calculation is done.

$\frac{N_0}{n}$ number of antibodies were produced at the instant T=0 and the number of antibodies present in the mouse at the instant T from the 1st production is $= \frac{N_0}{n} e^{-\lambda T}$.

Then, at the $\frac{T_R}{n}$ instant (counting from the same T=0 instant when the implant was inserted into the mouse), a 2nd installment of $\frac{N_0}{n}$ number of antibodies were produced in the mouse. Therefore, at the instant T, the number of antibodies present in the mouse from the 2nd production would be= $\frac{N_0}{n} e^{-\lambda\left(T-\frac{T_R}{n}\right)}$.

Similarly, the 3rd production of $\frac{N_0}{n}$ number of antibodies would take place at the instant $\frac{2T_R}{n}$ and the number of antibodies present in the mouse at the instant T from the 3rd production is $= \frac{N_0}{n} e^{-\lambda\left(T-\frac{2T_R}{n}\right)}$.

In this way, we can go on calculating up to the nth production. Hence, the number of antibodies present in the mouse at the instant T because of this slow release from the implant inserted in the mouse at the instant T=0 is

$$N_2(T) = \frac{N_0}{n} e^{-\lambda T} + \frac{N_0}{n} e^{-\lambda\left(T-\frac{T_R}{n}\right)} + \frac{N_0}{n} e^{-\lambda\left(T-\frac{2T_R}{n}\right)} + \cdots . + \frac{N_0}{n} e^{-\lambda\left[T-\frac{(n-1)T_R}{n}\right]}$$

$\therefore\ N_2(T) = \frac{N_0}{n} e^{-\lambda T} \sum_{k=0}^{k=n-1} e^{k\lambda\frac{T_R}{n}} = \frac{N_0}{n} e^{-\lambda T} \left(\frac{1-e^{\lambda T_R}}{1-e^{\lambda T_R/n}}\right)$ …………… (2)

(By summing the geometric progression series)

Then, we get

$$\frac{N_1(T)}{N_2(T)} = \frac{n\left(1-e^{\frac{\lambda T_R}{n}}\right)}{1-e^{\lambda T_R}}$$

Now, let us consider a continuous slow release with the linearity assumption as before. So, in the above equation, we have to take the limit $n \to \infty$.

Let us assume $x = \frac{1}{n}$ and we shall take the limit $x \to 0$. We rewrite the above equation as

$$\frac{N_1(T)}{N_2(T)} = \frac{\left(1-e^{x\lambda T_R}\right)}{x\left(1-e^{\lambda T}\right)}$$

So, for continuous slow release (using L'Hospital's theorem)

$\frac{N_1(T)}{N_2(T)} = \lim_{x\to 0} \frac{\left(1-e^{x\lambda T_R}\right)}{x\left(1-e^{\lambda T}\right)} = \frac{\lim_{x\to 0}\left(-\lambda T_R e^{x\lambda T_R}\right)}{\lim_{x\to 0}\left(1-e^{\lambda T_R}\right)} = \frac{-\lambda T_R}{1-e^{\lambda T_R}}$…………. (3)

So, at any instant T, $\frac{N_1(T)}{N_2(T)}$ is independent of T and depends only on λ and T_R. For HPV antibodies $T_R \approx 25$ days and the lifetime of the antibody is very long (>90 days) and hence $\lambda = \frac{1}{90}$ days^{-1}.

So, $\lambda T_R \ll 1$. Hence, from eq. (3), we get

$$\frac{N_1(T)}{N_2(T)} = \frac{-\lambda T_R}{1 - e^{\lambda T_R}} = \frac{-\lambda T_R}{1 - \left(1 + \lambda T_R + \frac{\lambda^2 T_R^2}{2} +..\right)} \approx \frac{-\lambda T_R}{-\lambda T_R} = 1$$

Hence, at any instant T, the ratio of antibodies in the mouse obtained from a single dose and slow release from an implant would be about the same.
